# Features of the cervicovaginal microenvironment drive cancer biomarker signatures in patients across cervical carcinogenesis

**DOI:** 10.1038/s41598-019-43849-5

**Published:** 2019-05-14

**Authors:** Paweł Łaniewski, Haiyan Cui, Denise J. Roe, Dominique Barnes, Alison Goulder, Bradley J. Monk, David L. Greenspan, Dana M. Chase, Melissa M. Herbst-Kralovetz

**Affiliations:** 1 Department of Basic Medical Sciences, College of Medicine-Phoenix, University of Arizona, Phoenix, AZ USA; 2 UA Cancer Center, University of Arizona, Tucson/Phoenix, AZ USA; 3 Maricopa Integrated Health Systems, Phoenix, AZ USA; 4 Dignity Health St. Joseph’s Hospital and Medical Center, Phoenix, AZ USA; 5 Department of Obstetrics and Gynecology, College of Medicine-Phoenix, University of Arizona, Phoenix, AZ USA; 6 US Oncology, Phoenix, AZ USA

**Keywords:** Microbiome, Mucosal immunology, Cancer microenvironment, Cervical cancer, Tumour biomarkers

## Abstract

Persistent human papillomavirus (HPV) infection is the vital factor driving cervical carcinogenesis; however, other features of the local cervicovaginal microenvironment (CVM) may play a critical role in development of precancerous cervical dysplasia and progression to invasive cervical carcinoma (ICC). Here we investigated relationships between locally secreted cancer biomarkers and features of the local CVM to better understand the complex interplay between host, virus and vaginal microbiota (VMB). We enrolled women with ICC, high- and low-grade squamous intraepithelial lesions, as well as, HPV-positive and healthy HPV-negative controls. A broad range of cancer biomarkers was present in the local CVM and specifically elevated in ICC patients. The majority of cancer biomarkers were positively correlated to other biomarkers and linked to genital inflammation. Several cancer biomarkers were also negatively correlated to *Lactobacillus* abundance and positively correlated with abnormal vaginal pH. Finally, a hierarchical clustering analysis of cancer biomarkers and immune mediators revealed three patient clusters, which varied in levels of cancer biomarkers, genital inflammation, vaginal pH and VMB composition. Specific cancer biomarkers discriminated patients with features of the CVM, such as high genital inflammation, elevated vaginal pH and dysbiotic non-*Lactobacillus*-dominant VMB, that have been associated with HPV persistence, dysplasia and progression to ICC.

## Introduction

Human papillomavirus (HPV) is the most common sexually transmitted infection worldwide, with an estimated 14.1 million new cases in the United States annually^1^. More than 200 HPV genotypes have been identified to date and over 40 genotypes are tropic to the genital mucosa and spread through sexual contact^2^. The majority of HPV infections are eliminated by the host within a few years. However, HPV infections that evade the immune system and are not cleared may lead to serious long-term health consequences. Many anogenital HPV types, defined as low-risk types, can cause benign abnormalities, i.e. genital warts. Conversely, high-risk HPV genotypes are well-established carcinogenic agents that can cause cervical, vulvar, penile, anal, and oropharyngeal tumors. Of these, cervical cancer represents the greatest global health burden, as it is the most common HPV-related cancer, with >500,000 new cases annually, and the fourth most common cancer in women^3^.

Ninety-nine percent of cervical cancers contain high-risk HPV^4^; however, the majority of high-risk HPV infections do not progress to cancer, but instead clear spontaneously. It is well documented that persistent HPV infection is the vital factor driving the development of precancerous cervical intraepithelial neoplasia (CIN) and progression to invasive cervical carcinoma (ICC)^5–10^. Several cofactors such as smoking, age of sexual debut, high parity, long-term use of contraceptives, hormone treatment and co-infections with sexually transmitted pathogens have been shown to be associated with progression of cervical neoplasia among HPV-infected women^11–13^. In recent years, accumulating evidence suggests that the microbiome might also play a significant role in the natural history of HPV infection^14–24^.

The vaginal microbiota (VMB) in the majority of healthy premenopausal women is dominated by *Lactobacillus* species (*Lactobacillus crispatus*, *Lactobacillus gasseri*, *Lactobacillus jensenii* or *Lactobacillus iners*)^25–28^. *Lactobacillus* spp. protect the host against genital infections through the production of lactic acid, which acidifies the local microenvironment, and by secretion of other antimicrobial compounds^28,29^. Consequently, women with *Lactobacillus*-dominant (LD) VMB exhibit low vaginal pH (≤4.5), an indicator of homeostasis and vaginal health^24,29^. When dysbiosis occurs, i.e. during bacterial vaginosis (BV), lactobacilli are depleted and replaced by a polymicrobial consortium of microaerophilic and anaerobic bacteria (*Gardnerella Atopobium*, *Prevotella*, *Sneathia*, *Anaerococcus*, *Peptostreptococcus*, *Parvimonas*, *Megasphaera*, and bacteria from other genera)^24,29^.

Recently, numerous epidemiological studies have shown associations between the dysbiotic non-*Lactobacillus*-dominant (NLD) VMB and HPV infection, development of precancerous dysplasia and progression to cervical cancer^14–24,30^. BV has been associated with a higher risk of HPV acquisition and decreased clearance of HPV^16–18^. Several reports also revealed that HPV-infected individuals more frequently have diverse NLD VMB^14,15^. Moreover, multiple clinical studies identified bacterial taxa associated with HPV persistence (*Atopobium*, *Mycoplasma hominis, Haemophilus*)^19,23,31,32^ and cervical neoplasia (*Sneathia, Atopobium, Parvimonas, Fusobacterium, Anaerococcus, Peptostreptococcus*)^20,21,24^. We and others also showed decreased *Lactobacillus* dominance and higher rates of diverse dysbiotic VMB in patients with precancerous lesions and cervical cancer, which correlated with increased vaginal pH^20,21,24^. Notably, one longitudinal study also found an association of *L. gasseri* with HPV clearance^19^.

To better understand the impact of VMB on the local cervicovaginal microenvironment (CVM), herein we examined a broad panel of known circulating cancer biomarkers (i.e. growth and angiogenic factors, hormones, pro-inflammatory cytokines/chemokines and others) in cervicovaginal lavages (CVL) collected from patients with cervical cancer or cervical dysplasia, as well as, healthy HPV-positive and HPV-negative women without neoplasia. Our comprehensive analysis allowed us to identify signatures associated with neoplastic disease and the VMB composition. Additionally, by integrating our cancer biomarker, immune mediator, vaginal pH and VMB datasets, we were able to reveal patient groups with particular features of CVM that may contribute to HPV persistence, development of precancerous lesions and progression to cancer. The presented study further investigates CVM features and brings us closer to understanding the complex relationship between the host, local microbiome and HPV during cervical carcinogenesis.

## Results

### Patient demographics, HPV status and vaginal pH

A total of 78 premenopausal women were classified into five groups: healthy HPV-negative controls (Ctrl HPV−; n = 18), HPV-positive controls (Ctrl HPV+; n = 11), women diagnosed with low-grade intraepithelial lesions (LSIL; n = 12), high-grade intraepithelial lesions (HSIL; n = 27) and ICC (n = 10). Table 1 shows the participant demographics. There was no significant difference among the groups in terms of age (*P* = 0.46), body mass index (BMI) (*P* = 0.97), HPV status (including HPV16, HPV18, high-risk and low-risk genotypes distribution) (*P* > 0.05), HPV risk profile (including infections with single or multiple high-risk genotypes as well as mixed infections with high-risk and low-risk genotypes) (*P* > 0.05). Prevalence of 37 HPV genotypes (including 13 high-risk and 24 low-risk genotypes) detected among the patient groups is shown in Supplementary Table 1. Forty-seven percent of patients were of Hispanic origin and 53% were of non-Hispanic origin. Hispanic ethnicity was not significantly different among the groups, ranging from 28% (5/18) in Ctrl HPV− to 63% (17/27) in HSIL (*P* = 0.15). Two factors, type of contraception and vaginal pH, varied across the groups. The vaginal pH data was associated with the disease severity, as the percentage of patients with abnormal pH (defined as >4.5) significantly varied (*P* = 0.003) and gradually increased among the patient groups (Table 1). Ctrl HPV− group had the lowest rate of abnormal pH (50%, 9/18), whereas ICC group had the highest rate of abnormal pH (100%, 9/9).

**Table 1:** Patient demographics.

	n	Ctrl HPV− (n = 18)	Ctrl HPV+ (n = 11)	LSIL (n = 12)	HSIL (n = 27)	ICC (n = 10)	*P* value
Age (mean (SD))	78	40.38 (6.98)	36.36 (9.53)	35.08 (7.24)	38.29 (8.46)	38.90 (9.09)	0.46
**Ethnicity** (**n** (**%))**							
Hispanic	37	5 (27.78)	4 (36.36)	7 (58.33)	17 (62.96)	4 (40.00)	0.15
Non-Hispanic	41	13 (72.72)	7 (63.64)	5 (41.67)	10 (37.04)	6 (60.00)	
**BMI** (**n** (**%))**							
≤25	25	7 (38.89)	3 (27.27)	4 (33.33)	8 (29.63)	3 (30.00)	0.97
>25	53	11 (61.11)	8 (72.73)	8 (66.67)	19 (70.37)	7 (70.00)	
**HPV status** (**n** (**%))**							
HPV16 positive	43		9 (81.82)	8 (72.73)	19 (70.37)	7 (70.00)	0.78
HPV18 positive	6		0 (0.00)	1 (9.09)	4 (14.81)	1 (10.00)	0.62
Other high-risk HPV	38		5 (45.55)	10 (83.33)	19 (70.37)	4 (40.00)	0.09
Low-risk HPV	2		0 (0.00)	0 (0.00)	1 (3.70)	1 (10.00)	0.45
**HPV risk profile** (**n** (**%))**							
Single high-risk	31		8 (72.73)	3 (25.00)	13 (48.15)	7 (70.00)	0.08
Multiple high-risk	26		3 (27.27)	8 (66.67)	13 (48.15)	2 (20.00)	0.11
High- and low-risk	57		11 (100.00)	11 (91.67)	26 (96.30)	9 (90.00)	0.55
**Type of contraception** (**n** (**%))**							
Hormonal	16	2 (20.00)	5 (62.50)	3 (42.86)	2 (11.11)	4 (80.00)	0.03
Non-hormonal	20	3 (30.00)	2 (25.00)	2 (28.57)	12 (66.67)	1 (20.00)	
None	12	5 (50.00)	1 (12.50)	2 (28.57)	4 (22,22)	0 (0.00)	
**Vaginal pH** (**n** (**%))**							
≤4.5	15	9 (50.00)	1 (11.11)	3 (27.27)	2 (7.41)	0 (0.00)	0.003
>4.5	59	9 (50.00)	8 (88.89)	8 (72.73)	25 (92.59)	9 (100.00)	

pH data available for 74 individuals; contraception data available for 48 individuals. *P* values were calculated using ANOVA for continuous variables and Fisher’s exact test for categorical values.

### Circulating cancer biomarkers are present in the local cervicovaginal microenvironment

**Figure 1:**
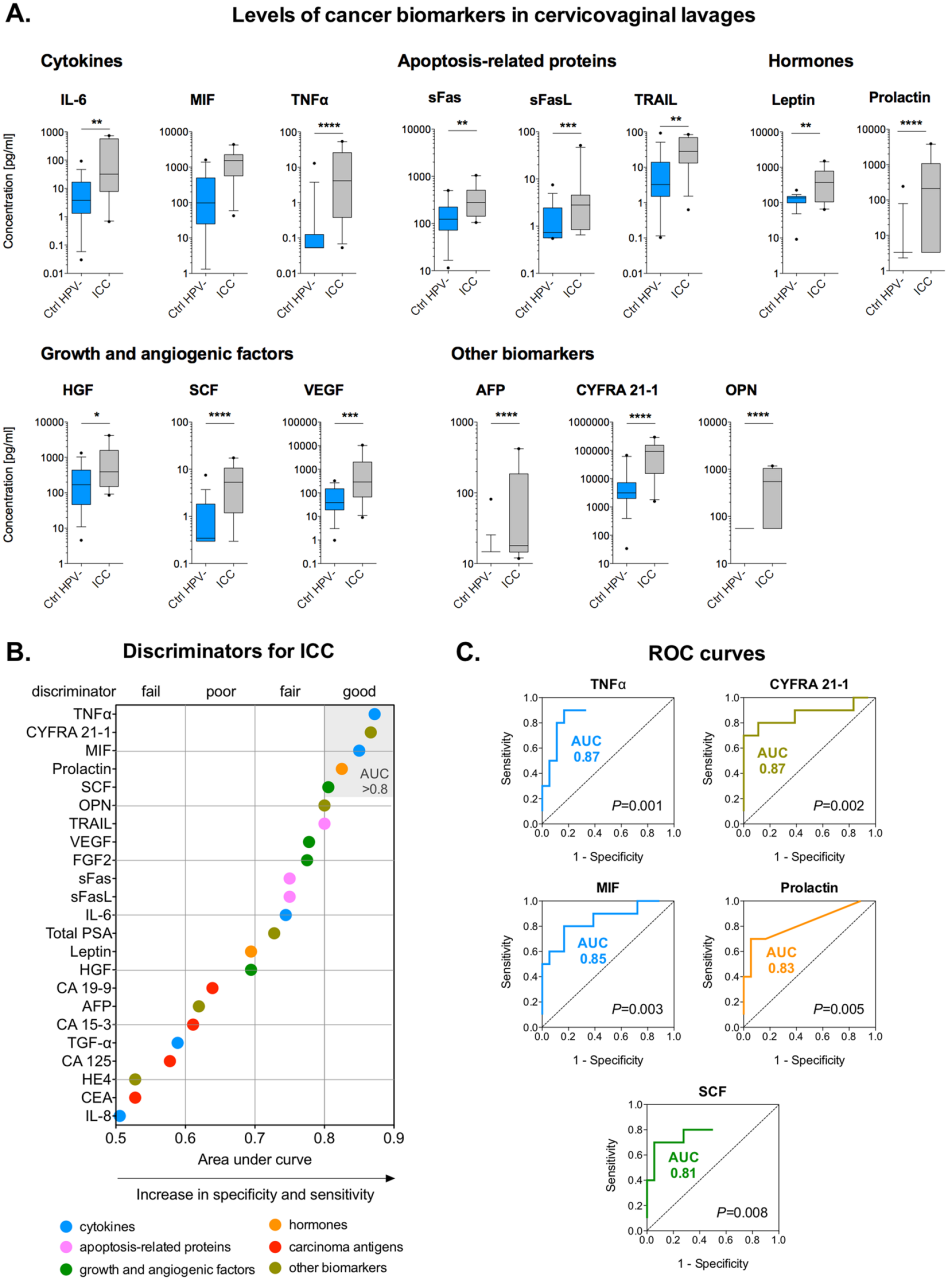
Cancer biomarkers are present in the cervicovaginal microenvironment and specifically elevated in cervical cancer patients. The level of cancer biomarkers in cervicovaginal lavages of ICC patients is compared to Ctrl HPV− (**A**). Box-and-whiskers plots represent the median and interquartile range with whiskers ranging between the 10^th^ and 90^th^ percentiles; dots indicate outliers. *P* values were calculated using linear mixed effects models where group was the fixed effect and replicate was the random effect with Tukey adjustment. *P < 0.05; **P < 0.01; ***P < 0.001; ***P < 0.0001. The receiver operating characteristics (ROC) analysis comparing ICC to Ctrl HPV− groups. Cancer biomarkers with area under curve (AUC) values greater than 0.8 serve as good discriminators for ICC (**B**). ROC curves indicating specificity and sensitivity of cancer biomarkers with AUC > 0.8 are depicted (**C**).

To characterize local cancer biomarkers in patients at various stages of cervical neoplasia, we measured 24 cancer biomarkers in CVL samples. The levels of 23 cancer biomarkers were in the detectable range; the levels of β-human chorionic gonadotropin (β-hCG) were below the detection limit. We found that 13 cancer biomarkers were significantly elevated in ICC (*P* ranging from 0.02 to <0.0001) when compared to Ctrl HPV− (Fig. 1A). Functionally, targets that were increased in ICC were comprised of proinflammatory cytokines [interleukin (IL)-6, tumor necrosis factor (TNFα)], apoptosis-related proteins [soluble Fas receptor (sFas), soluble Fas ligand (sFasL), TNF-related apoptosis-inducing ligand (TRAIL)], hormones (leptin, prolactin), growth and angiogenic factors [hepatocyte growth factor (HGF), stem cell factor (SCF), vascular endothelial growth factor (VEGF)] and other multi-functional proteins [osteopontin (OPN), cytokeratin fragment (CYFRA) 21-1, α-fetoprotein (AFP)]. Only levels of three biomarkers: HGF, OPN, transforming growth factor α (TGF-α) were altered in the precancerous groups (Supplementary Fig. S1). Levels of HGF and TGF-α were significantly decreased in HSIL (*P* = 0.001 and <0.05, respectively) and OPN levels were significantly increased in Ctrl HPV+ (*P* = 0.04) when compared to Ctrl HPV−. Additionally, we performed a receiver operating characteristics (ROC) analysis to evaluate discriminatory abilities for these cancer biomarkers (Supplementary Fig. S2). Cancer biomarkers with area under curve (AUC) values, which indicate both sensitivity and specificity, greater than 0.8 were considered as good discriminators and 0.9 as excellent discriminators. The ROC analysis comparing patients with ICC to Ctrl HPV− revealed that five tested cancer biomarkers: TNFα, CYFRA 21-1, macrophage migration inhibitory factor (MIF), prolactin and SCF had AUC greater than 0.8 (Fig. 1B,C). This analysis demonstrated that these five cancer biomarkers, when measured in the CVL samples, can significantly distinguish ICC patients from healthy Ctrl HPV−. These novel findings demonstrate that circulating cancer biomarkers that are normally detectable in the plasma/serum can also be found in the local CVM and are specifically elevated in cervical cancer patients, but not in patients with precancerous lesions or healthy controls.

### Cancer biomarkers are related to cervical carcinoma and linked to genital inflammation and microbiota composition

**Figure 2:**
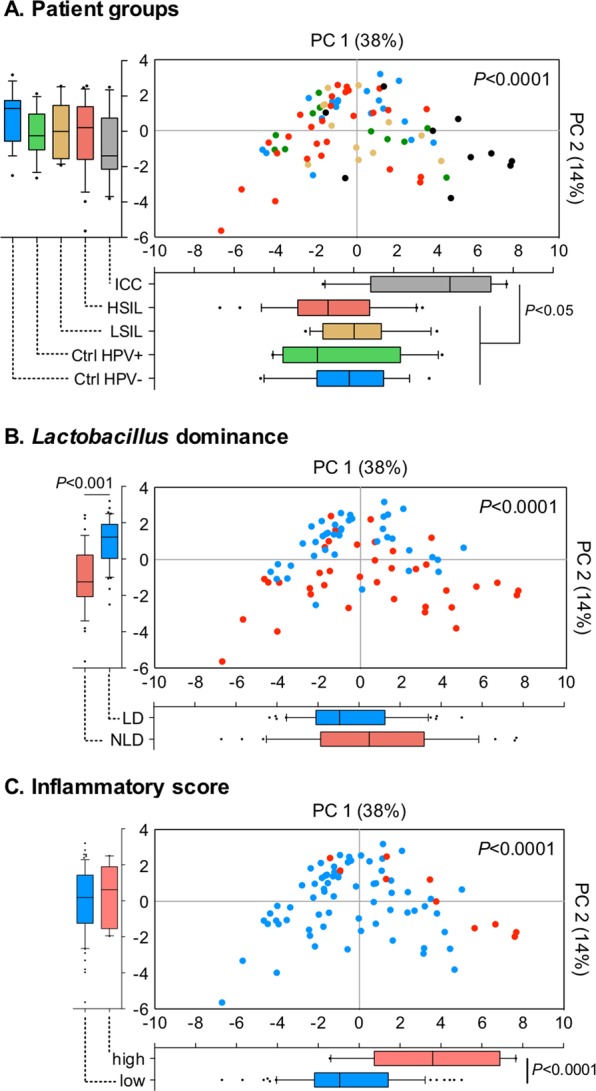
Cancer biomarkers cluster according to disease severity, vaginal microbiota composition and level of genital inflammation. Principal component analysis of all cancer biomarker data displayed along the first two principal components, with each point representing a single sample colored according to patient group (**A**), *Lactobacillus* dominance (defined as ≥80% relative abundance) (**B**), or inflammatory score (low inflammatory score: 0–4; high inflammatory score: 5–7) (**C**). Box-and-whiskers plots shown along each principal component axis represent the median and interquartile range with whiskers ranging between the 10^th^ and 90^th^ percentiles and indicate the distribution of samples along the given axis. *P* values were calculated using MANOVA.

To reduce dimensionality of the data, we applied a principal component analysis (PCA) for the measured cancer biomarker levels (Fig. 2). The first two principal components explained 52.0% of the variance in the data and were used in our analysis. The analysis revealed that samples from patients with ICC significantly separated (*P* < 0.0001) from samples collected from other patients, including healthy controls as well those with precancerous dysplasia (Fig. 2A). Pairwise comparisons demonstrated statistically significant differences between ICC and Ctrl HPV− (*P* = 0.0001), ICC and Ctrl HPV+ (*P* = 0.002), and ICC and LSIL (*P* = 0.004), as well as, ICC and HSIL (*P* = 0.0001). Analysis of each component separately showed that only the first principal component, but not the second principal component, was significantly different among the patient groups (Fig. 2A). The analysis revealed that expression of cancer biomarkers in the local CVM is related to cervical carcinoma.

In our previous report, we showed significant changes in the VMB composition (decrease in *Lactobacillus* dominance) and increased evidence of genital inflammation in patients with ICC^24^, therefore we included these factors in the principal component analysis to determine if these factors were associated with cancer biomarker patterns. For *Lactobacillus* dominance, samples were subdivided into two categories: samples from patients with LD microbiota (relative abundance ≥80%)) versus samples from patients with NLD microbiota (relative abundance <80%). For evidence of genital inflammation, samples were subdivided into two groups: samples from patients with high inflammatory scores (5–7) and samples from patients with low inflammatory scores (0–4). The inflammatory score for each patient was determined previously by assigning one point for each cytokine/chemokine: IL-1α, IL-1β, IL-8, macrophage inflammatory protein (MIP)-1β, MIP-3α, RANTES, and TNFα, when its level was in the upper quartile^24^. The PCA of cancer biomarkers showed that samples also significantly cluster according to *Lactobacillus* dominance (*P* < 0.0001; Fig. 2B) and inflammatory score (*P* < 0.0001; Fig. 2C). When the first and second principal components were analyzed individually, LD and NLD groups were significantly different only in the second principal component (*P* < 0.001), whereas low (score: 0–4) and high inflammatory score (score: 5–7) groups were significantly different in the first principal component (*P* < 0.0001). The PCA also showed that samples did not group into separate clusters according to ethnicity, vaginal pH or BMI (data not shown). These data illustrate that cancer biomarker expression is also linked to the level of genital inflammation and the VMB composition.

### The majority of cancer biomarkers are strongly correlated with other cancer biomarkers

**Figure 3:**
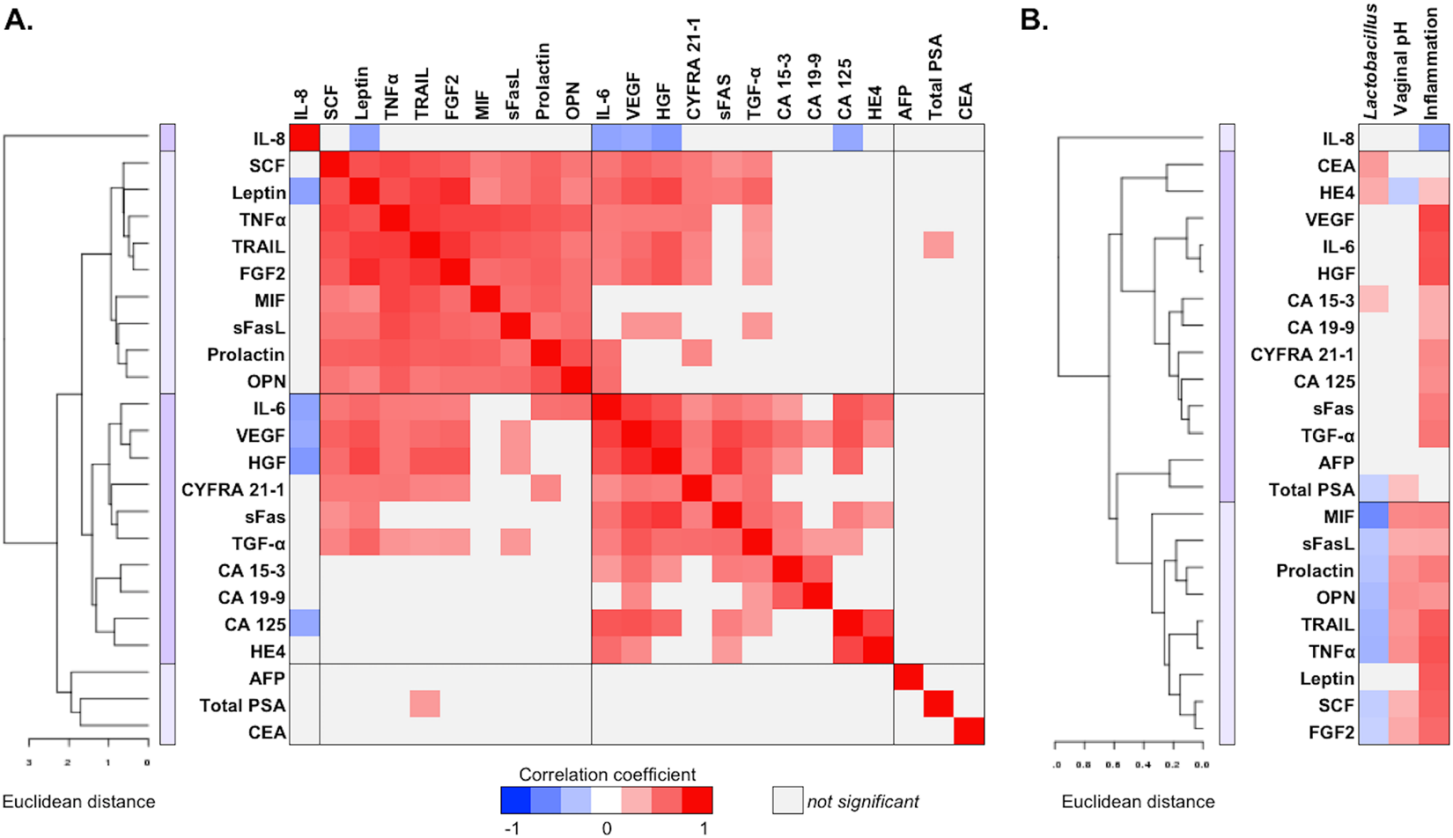
Cancer biomarkers are strongly correlated to other cancer biomarkers, genital inflammation, as well as, vaginal pH and *Lactobacillus* abundance. Correlation of cancer biomarkers to other cancer biomarkers (**A**), or *Lactobacillus* abundance, vaginal pH and inflammatory scores (**B**) in the cervicovaginal lavages among all the patients are depicted. Correlation coefficients (ρ) were calculated using Spearman’s rank correlation analysis. Hierarchical clustering of correlation coefficients was performed using CIMminer based on Euclidean distance and average linkage cluster algorithm. A bar on each dendrogram indicates separate clusters of cancer biomarkers. Only correlation coefficients that were significant (*P* < 0.05) are displayed on the heat map. Red- and blue-shaded squares indicate positive and negative correlations, respectively. All correlation coefficient values can be found in Supplementary Figs S3 and S4.

To explore the correlation of each cancer biomarker to other cancer biomarkers in the local CVM, we analyzed concentration levels of cancer biomarkers in all tested samples, regardless of patient groups. We computed Spearman’s correlation coefficients (ρ) for all pairs of cancer biomarkers (Fig. 3A and Supplementary Fig. S3). The analysis showed that most cancer biomarkers in the CVL samples were positively correlated, which is evidenced by the majority of red-shaded squares of the heat map [only coefficients that were significant (*P* < 0.05) are depicted on the heat map] (Fig. 3A). We noted that after excluding self-to-self correlation, 40.3% of all pairs of cancer biomarkers (102/253) exhibited a positive correlation (adjusted *P* < 0.05). Hierarchical clustering analysis of the correlation matrix also revealed two major groups of cancer biomarkers strongly correlated to each other. The first group consisted of SCF, leptin, TNFα, TRAIL, basic fibroblast growth factor (FGF2), MIF, sFasL, prolactin and OPN and the second group consisted of IL-6, VEGF, HGF, CYFRA 21-1, sFas, TGF-α, carcinoma antigen (CA) 15-3, CA 125 and human epididymis protein 4 (HE4). In the first cluster, 100% of pairs of cancer biomarkers (36/36) exhibited positive correlation (adjusted *P* < 0.05) with the highest correlations between FGF2 and leptin (ρ = 0.84, *P* < 0.0001), FGF2 and TRAIL (ρ = 0.80, *P* < 0.0001) and leptin and TRAIL (ρ = 0.77, *P* < 0.0001). In the other cluster, 71.1% of pairs of cancer biomarkers (32/45) exhibited positive correlation (*P* < 0.05) with the highest correlations between HGF and VEGF (ρ = 0.84 *P* < 0.0001), HGF and sFas (ρ = 0.79, *P* < 0.0001) and IL-6 and VEGF (ρ = 0.76, *P* < 0.0001). The analysis showed that women with a high concentration of one cancer biomarker were relatively likely to have high concentrations of other cancer biomarkers.

### Cancer biomarkers are correlated to genital inflammation, vaginal pH and *Lactobacillus* abundance

Since the PCA analyses showed that genital inflammation and vaginal microbiota composition impact cancer biomarker signatures, we also examined correlations between the levels of cancer biomarkers (in all tested samples regardless of patient groups) and inflammatory scores, *Lactobacillus* abundance or vaginal pH (Fig. 3B and Supplementary Fig. S4). The analysis revealed that 19 out of 23 cancer biomarkers (82.61%) exhibited significant positive correlation to inflammatory scores (adjusted *P* < 0.05). Furthermore, nine cancer biomarkers: MIF, TNFα, sFasL, TRAIL, FGF2, SCF, prolactin and OPN, were negatively correlated to *Lactobacillus* abundance and positively correlated to levels of vaginal pH. Interestingly, we also identified that an antimicrobial peptide, HE4, to be positively correlated to *Lactobacillus* abundance and negatively correlated to vaginal pH. Additionally, we examined associations among secreted cancer biomarkers, patient groups, and *Lactobacillus* dominance (relative abundance ≥80%) using mixed effects model (Supplementary Fig. S5). As other factors might also contribute to changes in the microbiota composition, we controlled our data for age, BMI and ethnicity. Following adjustment for covariates, only three cancer biomarkers, CYFRA 21-1, SCF and OPN, were positively associated with ICC in patients with LD or NLD microbiota, whereas eleven cancer biomarkers, IL-6, TNFα, TRAIL, sFas, sFasL, FGF2, HGF, VEGF, prolactin, leptin, and AFP were positively associated with ICC only in patients with NLD microbiota when compared to Ctrl HPV−. The analysis further confirmed that associations of cancer biomarkers with invasive carcinoma depend on the VMB composition, specifically non-*Lactobacillus* dominance.

### Hierarchical clustering of cancer biomarkers reveals high- and low-risk clusters associated with features of cervicovaginal microenvironment

**Figure 4:**
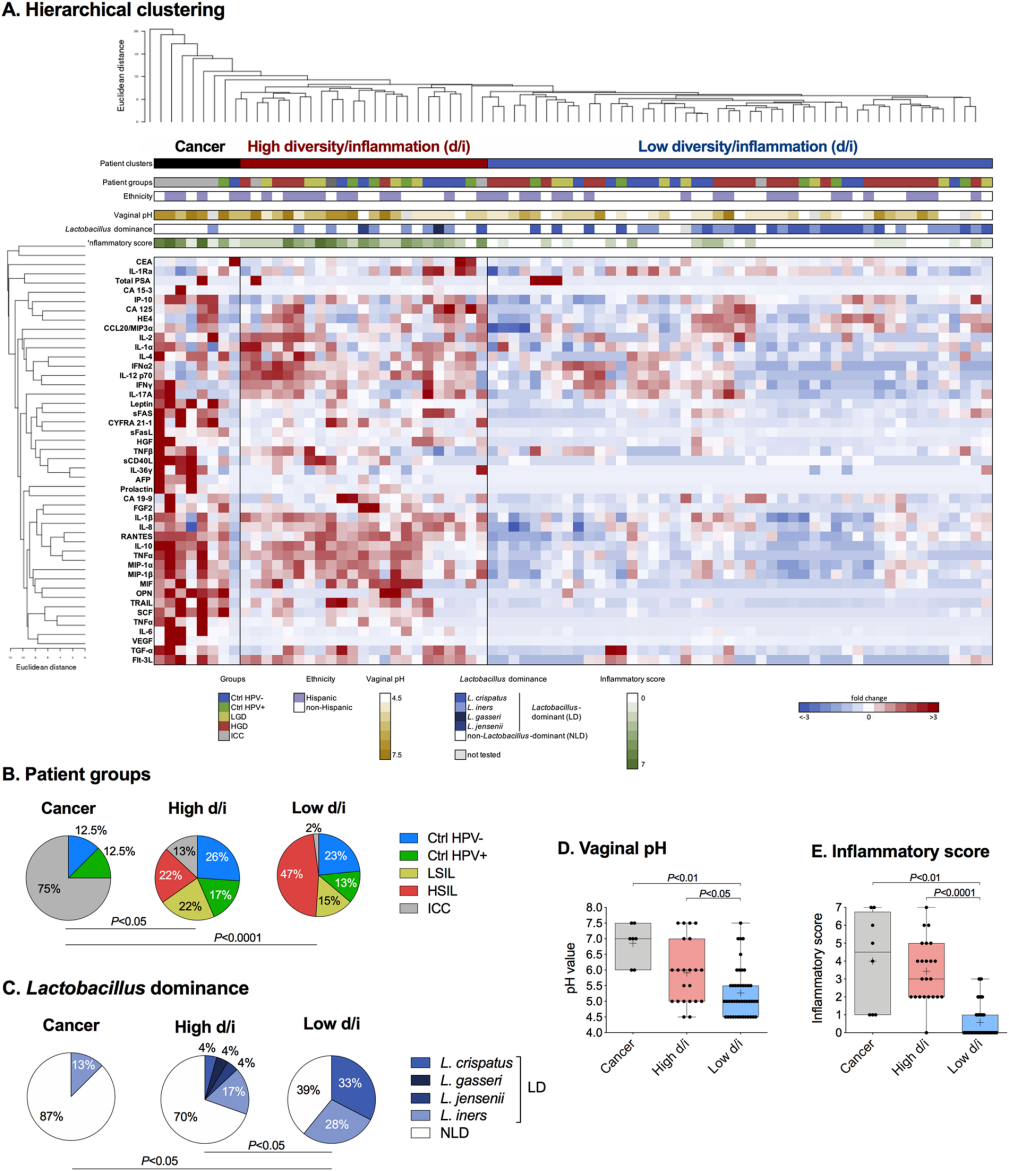
Hierarchical clustering analysis reveals three distinct patient clusters: cancer-associated, high diversity/inflammation and low diversity/inflammation. A heat map reflects relative levels of cancer biomarkers and immune mediators in the cervicovaginal lavages across all the samples. Data were mean centered and variance scaled along each row before clustering. Hierarchical clustering was performed using CIMminer based on Euclidean distance between rows and columns and the average linkage cluster algorithm. Red- and blue-shaded squares indicate increased or decreased levels compared to the mean value for each target, respectively. Patient clusters, patient groups, ethnicity, vaginal pH, *Lactobacillus* dominance, pre-dominant *Lactobacillus* species and inflammation score are also shown across above the heat map (**A**). Distribution of patient groups (**B**), *Lactobacillus* dominance (**C**) and levels of vaginal pH (**D**) and inflammatory scores (**E**) were significantly different among the clusters (cancer: cancer-associated cluster; high d/i: high diversity/inflammation cluster; low h/i: low diversity/inflammation cluster). *P* values were calculated using Kruskal-Wallis or Fisher’s exact test.

To investigate the complex interplay between local cancer biomarkers, cytokine/chemokines and patient-specific factors we performed unsupervised hierarchical clustering analysis of cancer biomarker data together with immune mediator data. The heat map and dendrogram revealed three distinct clusters of patients (Fig. 4A). To characterize these clusters, we plotted metadata available for each patient (patient group/disease severity, ethnicity, vaginal pH, *Lactobacillus* dominance and inflammatory score) above the heat map and analyzed statistical differences in those patient-specific factors among three clusters. We found that distribution of patient groups, rates of *Lactobacillus* dominance, levels of vaginal pH and inflammatory scores were significantly different among the clusters (*P* < 0.001). The percentage of patients of Hispanic origin, compared to non-Hispanic origin, did not vary among the clusters (*P* = 0.75). Based on the characteristics described below, we designated the patient clusters as associated with cancer, associated with high VMB diversity and high inflammation and associated with low VMB diversity and low inflammation. First, the distribution of patient groups significantly differed among the three clusters (*P* = 0.0002) (Fig. 4B). The cancer-associated cluster had the highest rate of patients with ICC (75.00%), whereas the high and low diversity/inflammation clusters consisted of only 13% and 2% patients with ICC, respectively. Interestingly, the high and low diversity/inflammation clusters did not significantly vary based on disease severity (*P* = 0.15). However, rates of patients with dysbiotic NLD microbiota (defined as <80% relative abundance of *Lactobacillus* spp.) were significantly increased in the cancer-associated cluster (87.50%; *P* = 0.02), as well as, the high diversity/inflammation cluster (69.57%; *P* = 0.01) when compared to the low diversity/inflammation cluster (38.87%). Furthermore, for the patients with LD microbiota, the predominant *Lactobacillus* species also varied among the clusters (*P* < 0.05). Vaginal health-associated *L. crispatus* commonly predominated patients’ microbiota in the low diversity/inflammation cluster (32.61% of patients), but not in the high diversity/inflammation or cancer-associated clusters (4.35% and 0.00%, respectively), whereas intermediate *L. iners* were found among all patient clusters. Mean pH values were also significantly different among the clusters (*P* = 0.0002) **(**Fig. 4D). Vaginal pH data were available for 74/78 women. Vaginal pH was significantly elevated in the cancer-associated cluster (mean ± SD 6.85 ± 0.62; *P* = 0.001), and high diversity/inflammation cluster (mean ± SD 5.91 ± 1.01) compared to the low diversity/inflammation cluster (mean ± SD 5.26 ± 0.78; *P* = 0.02). Similarly, inflammatory score, which is an evidence of genital inflammation, varied significantly among the clusters (*P* < 0.0001) (Fig. 4E). The cancer-associated cluster had the highest inflammatory score (mean ± SD 4.00 ± 2.67), whereas the low diversity/inflammation cluster had the lowest score (mean ± SD 0.57 ± 0.92), which was statistically significant (*P* = 0.0002). The high diversity/inflammation cluster also had a significantly elevated inflammatory score (mean ± SD 3.42 ± 1.70) compared to the low diversity/inflammation cluster (*P* < 0.0001).

**Figure 5:**
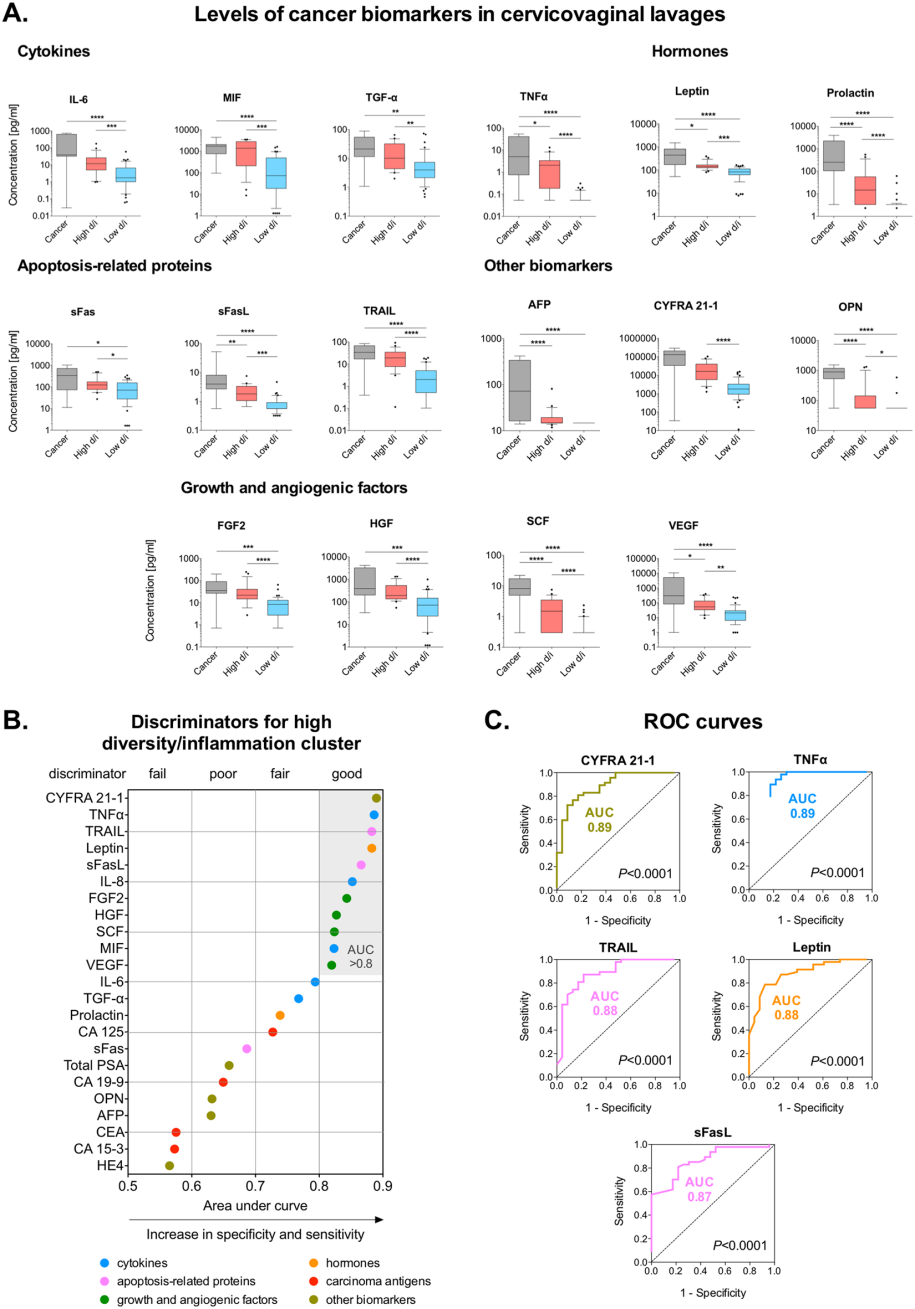
Several cancer biomarkers strongly discriminate cancer-associated, high diversity/inflammation and low diversity/inflammation cluster groups, which differ in vaginal pH, microbiota composition and genital inflammation. The level of cancer biomarkers in cervicovaginal lavages among the three cluster groups from the hierarchical clustering analysis (**A**). Box-and-whiskers plots represent the median and interquartile range with whiskers ranging between the 10^th^ and 90^th^ percentiles; dots indicate outliers. *P* values were calculated using linear mixed effects models where group was the fixed effect and replicate was the random effect with Tukey adjustment. *P < 0.05; **P < 0.01; ***P < 0.001; ***P < 0.0001. The receiver operating characteristics (ROC) analysis comparing the high diversity/inflammation (d/i) cluster to the low diversity/inflammation (d/i) cluster. Cancer biomarkers with area under curve (AUC) values greater than 0.8 serve as good discriminators for the high diversity/inflammation cluster group (**B**). ROC curves indicating specificity and sensitivity of cancer biomarkers with AUC > 0.85 are depicted (**C**).

We also compared the levels of tested cancer biomarkers in CVL samples among the three clusters (Supplementary Fig. S6). We found that 15 cancer biomarkers were significantly elevated in both high diversity/inflammation and cancer-associated clusters (*P* ranging from 0.03 to <0.0001) when compared to the low diversity/inflammation cluster (Fig. 5A). Notably, these cancer biomarkers functionally comprised of several cytokines (IL-6, MIF, TGF-α, TNFα), apoptosis-related proteins (sFas, sFasL, TRAIL), growth and angiogenic factors (FGF2, HGF, SCF, VEGF), hormones (leptin, prolactin) and other biomarkers (AFP, OPN), but not carcinoma antigens. Seven of these biomarkers (TNFα, sFasL, SCF, VEGF, leptin, prolactin, OPN) were also significantly elevated in the cancer-associated cluster compared to the high diversity/inflammation cluster (*P* ranging from 0.02 to <0.0001) (Fig. 5A). Furthermore, we performed the ROC analysis comparing the high and low diversity/inflammation clusters (Supplementary Fig. S7). The analysis revealed that 11 targets have good discriminatory potential (AUC > 0.8) between the high and low diversity/inflammation clusters (Fig. 5B). The biomarkers with the highest sensitivity and specificity were CYFRA 21-1, TNFα, TRAIL, leptin and sFasL (AUC 0.87–0.89, *P* < 0.0001) (Fig. 5C). Interestingly, a subset of identified cancer biomarkers (TNFα, sFasL, TRAIL, prolactin, FGF2, SCF, OPN), which were identified in the high diversity/inflammation cluster, were also correlated with *Lactobacillus* abundance, vaginal pH and genital inflammation (Fig. 3B) and were elevated in the ICC group (Fig. 1A).

Overall, these data allowed us to stratify patients into three groups: (i) the cancer-associated group, (ii) the high diversity/inflammation group, and (iii) the low diversity/inflammation group. Both the cancer-associated group and the high diversity/inflammation group were characterized by elevated levels of locally secreted cancer biomarkers, low rates of *Lactobacillus* dominance, high vaginal pH and high genital inflammation, all features associated with HPV persistence and hallmarks of cancer^14,15,19–21,23,24,33,34^. In contrast, the low diversity/inflammation group was characterized by a high abundance of *Lactobacillus*, lower vaginal pH and low genital inflammation, all features associated with vaginal health^19,33^.

## Discussion

Persistent infection with high-risk HPV is a well-established risk factor for development of ICC and its precancerous precursor, CIN. However, the majority of HPV infections are cleared by the host. Thus, other features of the local CVM (e.g. microbiome, pH, host immune mediators, environmental factors) may play a critical role in HPV persistence and impact hallmarks of cancer. In this report, we investigated relationships between locally secreted cancer biomarkers, VMB, vaginal pH, genital inflammation, and the severity of cervical neoplasia to determine features of the CVM that may contribute to HPV persistence, development of dysplasia and progression to cancer.

To our knowledge, this is the first report comprehensively examining the level of circulating cancer biomarkers in the local CVM. Herein, we took an unbiased approach and screened a broad range of proteins that are involved in neoplastic transformation. We determined levels of 24 targets in CVL samples collected from patients with ICC, HSIL, and LSIL, defined by histology, as well as, HPV-positive and HPV-negative women without neoplasia. These biomarkers are proteins that play a vital role during tumor development and progression, but also exhibit physiological functions (apoptosis-related proteins, growth factors, etc.) during homeostasis.

Our analysis revealed elevated levels of several biomarkers, including proinflammatory cytokines (IL-6, TNFα), apoptosis-related proteins (sFas, sFasL, TRAIL), hormones (leptin, prolactin), growth and angiogenesis factors (HGF, SCF, VEGF), cytokeratins (CYFRA 21-1), and others (AFP, OPN), only in cervical cancer patients, but not in other patient groups. Moreover, the biomarker discovery analysis showed that several of these biomarkers (TNFα, CYFRA 21-1, MIF, prolactin and SCF) exhibit high sensitivity and specificity for ICC when measured in CVL samples. A very limited number of studies have examined cancer biomarkers in cervicovaginal secretions [IL-6, IL-8, CA 125, carcinoembryonic antigen (CEA), and squamous cell carcinoma antigen (SCC-Ag)]^35–37^, although numerous reports showed that several of these biomarkers were significantly elevated in serum and/or biopsies collected from patients with invasive cervical carcinoma. Recent studies have also utilized cellular microRNA, single nucleotide polymorphism and DNA methylation patterns or gene/protein expression profiles as potential detection and prognosis markers^38–41^. Our data confirmed findings from a previous study reporting elevated levels of IL-6 in CVL of cervical cancer patients when compared to healthy controls or patients with cervical dysplasia^35^. Similar to our CVL findings, other researchers found significantly increased levels of IL-6, VEGF, HGF and OPN in both cancer patients’ sera and cancerous tissues^42–49^. Other immunohistochemical studies also showed overexpression of FasL, TRAIL and leptin in cancerous tissues^50^, which was consistent with our CVL findings. In contrast to our results, a study by McDicken *et al*. showed elevated levels of CEA in CVL of patients with CIN and ICC compared to controls^36^. However, another study suggested that CEA is a normal constituent of vaginal fluid, with levels that may be affected, not only by cancer, but also pregnancy or genital inflammation^37^. Ascencio-Cedillo *et al*. also showed that there was no difference in expression of prolactin in cancer tissue or serum when compared to samples collected from healthy individuals^51^, which we found to be elevated in CVL. Other serum biomarkers, which have been previously used for cervical cancer diagnosis and prognosis, included SCC-Ag, CA 125, CA 15-3, CEA and CYFRA 21-1^41,52^. We did not test SCC-Ag in this study, nor did we observe elevated levels of CA 125 or CA 15-3 in CVL in patients with ICC. Herein, we confirmed that a broad range of circulating cancer biomarkers are present in the local CVM and specifically elevated in women with ICC. It is important to note that local and systemic levels of these proteins may significantly differ, which might reflect their distinct functions during homeostasis and during neoplastic transformation. Specificity of these proteins as biomarkers may also be dependent on protein localization.

In our study, we also found that the majority of cancer biomarkers were highly correlated to other cancer biomarkers, which indicates that these proteins form biological integration networks in the local CVM. Using correlation and hierarchical clustering analyses, we identified two groups of cancer biomarkers highly correlated with each other. The first group was comprised of IL-6, VEGF, HGF, CYFRA 21-1, sFas, TGF-α, CA 15-3, CA 125, and HE4, with the highest correlations between HGF, VEGF and IL-6. It is well documented that IL-6 plays a central role in tumor progression due its ability to promote cell proliferation and inhibit apoptosis^53^. Both *in vitro* and *in vivo* studies demonstrated that high local IL-6 levels can stimulate VEGF production and promote tumor angiogenesis, and development of cervical cancer^47,54^. In our study we observed that patients concomitantly exhibited high levels of IL-6 and VEGF in CVL. Moreover, we also observed a strong positive correlation of VEGF and HGF, which indicates that HGF plays an important role during cervical carcinogenesis. Studies have reported that c-Met activation by HGF stimulates multiple signal transduction pathways, which results in cell proliferation, motility, tumor progression, and metastasis^55^. The other cluster consisted of leptin, FGF2, TNFα, MIF, TRAIL, SCF, sFasL, prolactin, and OPN, with the highest correlation between leptin and FGF2. Leptin, which is one of the main adipokines secreted by adipose tissue, has been implicated in the progression of multiple cancers through activation of the Janus kinase 2 (JAK)-signaling transducer and activator of transcription (STAT)-3 signaling pathway^56^. Leptin regulates cell turnover and facilitates the progression of cervical cancer^57^. FGF2 is similar to VEGF and acts as a potent angiogenic growth factor^58^. A study by Cao *et al*. has shown that leptin induces vascular permeability and acts synergistically with VEGF and FGF2 to promote angiogenesis^59^.

In this report, we integrated multiple datasets (immune mediators, genital inflammation, VMB composition, and vaginal pH) with the cancer biomarker data to perform exploratory data analysis using unsupervised methods. Using data reduction analyses we revealed that local cancer biomarker secretion is associated with both genital inflammation and the VMB composition. Our analyses revealed that the levels of majority of cancer biomarkers correlated with genital inflammation. We also identified a subset of cancer biomarkers that strongly correlated, not only with genital inflammation, but also with *Lactobacillus* abundance and vaginal pH, suggesting that these features of CVM may play a crucial role during neoplastic transformation. The data integration allowed us to stratify patients into distinct groups characterized by CVM features, such as high VMB diversity and inflammation, that may promote HPV persistence and cancer/dysplasia progression. As expected, the hierarchical clustering analysis of cancer biomarkers and immune mediators showed that the majority of cancer patients formed a separate cluster, that we assigned the “cancer-associated group”. However, the hierarchical clustering analysis revealed little stratification of patients based on either the severity of precancerous lesions (HSIL, LSIL) or HPV status (HPV-positive and HPV-negative controls) in the other two clusters. Instead, we observed two patient clusters, assigned as “high diversity/inflammation” and “low diversity/inflammation” groups, based on features of the CVM (genital inflammation, level of vaginal pH and the VMB composition). The cancer-associated and high-diversity/inflammation groups were both characterized by low levels of *Lactobacillus* dominance, high vaginal pH and high genital inflammation and elevated levels of cancer biomarkers, known to be associated with HPV persistence, development of dysplasia and progression to cancer^14,15,19–21,23,24,33,34^. In contrast, the low diversity/inflammation group was characterized by high levels of *Lactobacillus* dominance, lower vaginal pH, low genital inflammation, and low levels of cancer biomarkers, all features associated with vaginal health^19,33^. Remarkably, several cancer biomarkers (TNFα, sFasL, TRAIL, prolactin, FGF2, SCF, OPN), which were elevated in the cancer-associated and high diversity/inflammation clusters when compared to the low diversity/inflammation cluster, were also significantly and negatively correlated with *Lactobacillus* abundance and positively correlated with abnormal vaginal pH and genital inflammation. Furthermore, a subset of cancer biomarkers (CYFRA 21-1, TNFα, TRAIL, leptin and sFasL) strongly discriminated the high and low diversity/inflammation cluster groups. The role of these proteins in cervical carcinogenesis warrants further investigation, particularly in the context of vaginal microbiome and/or chronic inflammation. Future studies may investigate the potential use of these proteins as markers for disease progression and prognosis of therapeutic outcomes.

In our previous report, we showed that patients with cervical cancer exhibit an increased level of genital inflammation^24^. We hypothesize that local chronic inflammation, a hallmark of cancer, may suppress anti-tumor immunity and favor tumor progression. Numerous studies have demonstrated that development of HPV-induced cervical cancer is preceded by chronic inflammation^53^. However, in the majority of reports, genital inflammation has been due to the presence of concurrent infection with other sexually transmitted pathogens, including herpes simplex virus 2 and *Chlamydia trachomatis*, or other non-specific infections, including BV^16–18,60–62^. Recent studies have revealed that in women without other sexually transmitted infections, HPV infection or clearance is not associated with genital inflammation, but rather the VMB composition^60,63^. We and others also have shown association between the dysbiotic NLD VMB and HPV infection and the severity of cervical neoplasia^14–21,23,24,30^. Abnormal vaginal pH, which correlates with dysbiotic VMB composition, has been also associated with cervical neoplasia^24^. These microenvironmental factors, such as, genital inflammation, abnormal vaginal pH and diverse NLD VMB, coupled with increased levels of cancer biomarkers, may favor HPV persistence in the local microenvironment and consequently increase the risk of neoplastic disease. Alternatively, the collective action of these conditions, including infection with high-risk HPV, may directly impact hallmarks of cancer (cell proliferation, evasion of apoptosis, senescence, DNA mutation and methylation, and angiogenesis), contributing to development of precancerous lesions and progression to cancer.

The limitations of our study include a modest sample size, the lack of specific sexual behavior (i.e. number of sexual partners or age of sexual debut), smoking history data and a cross-sectional study design, showing associations, not causation. In the future, a larger cohort and longitudinal clinical studies will be needed to extend our findings for potential clinical utility. Moreover, mechanistic studies using *in vitro* and *in vivo* models, will be required to fully understand the complex relationships in the CVM between HPV, VMB, genital inflammation and development/progression of neoplastic disease.

In conclusion, we showed that a broad range of circulating cancer biomarkers is present in the local CVM and identified several cancer biomarkers that were specifically elevated in cervical cancer patients. We also revealed that the majority of cancer biomarkers exist concomitantly, which suggests that these proteins form biological interaction networks in the local microenvironment. Furthermore, we demonstrated that expression of cancer biomarkers is correlated not only with genital inflammation, but also vaginal microbiota composition (i.e. *Lactobacillus* abundance) and vaginal pH. By integrating our cancer biomarker, immune mediator, vaginal pH, and VMB datasets, we revealed three patient clusters: cancer associated, high diversity/inflammation and low diversity/inflammation. Finally, we identified several cancer biomarkers that could further delineate patients with dysbiotic VMB and a high level of genital inflammation from patients with *Lactobacillus*-dominant VMB and a low level of genital inflammation, which may be valuable predictors for HPV persistence, developing dysplasia and progressing to invasive carcinoma.

## Methods

### Study population

Patients were recruited at three clinical sites located in Phoenix, AZ: St. Joseph’s Hospital and Medical Center, University of Arizona Cancer Center and Maricopa Integrated Health Systems. All patients provided informed written consent and all research and related activities involving human subjects were approved by the Institutional Review Boards at Dignity Health St. Joseph’s Hospital and Medical Center, University of Arizona, and Maricopa Integrated Health Systems and performed in accordance with federal guidelines and regulations and the Declaration of Helsinki.

Samples collected from 78 premenopausal, non-pregnant, women diagnosed with ICC (n = 10), HSIL (n = 27) and LSIL (n = 12), as well as, healthy Ctrl HPV+ (n = 11) and Ctrl HPV− (n = 18) were included in this study. Classification of patients into the five groups and exclusion criteria were described previously^24^. Briefly, histology of colposcopy-directed biopsy samples was used to classify patients into groups. If histology was not available (i.e. for healthy Ctrl HPV+ and Ctrl HPV−), cytology was used for classification. Exclusion criteria included: currently menstruating; currently on antibiotics, antifungals or antivirals or within the previous three months; current vaginal infection (including BV), vulvar infection, urinary tract infection or sexually transmitted infection (chlamydia, gonorrhea, trichomoniasis, genital herpes, human immunodeficiency virus) or within the previous three weeks; unusual or foul-smelling vaginal discharge; use of douching substances, vaginal applied medications and suppositories, feminine deodorant sprays, vaginal lubricants within 48 hours prior the visit; any skin condition in the genital area interfering with the study; sexual intercourse less than 48 hours prior the visit; type I or type II diabetes; hepatitis.

### Sample collection and processing, HPV genotyping, 16S rRNA sequencing, multiplex analysis of immune mediators and genital inflammatory score system

Collection and processing of vaginal swabs, vaginal pH and CVL samples were described previously^24^. Briefly, mid vaginal swabs were collected by a physician using the eSwab collection system with Amies transport medium (COPAN, Diagnostics, Murrieta, CA); vaginal pH was measured using nitrazine paper; CVL were collected using 10 ml of sterile 0.9% saline solution. Collected samples were immediately placed on ice and frozen at −80 °C, thawed on ice and aliquoted to avoid multiple freeze/thaw cycles and stored at −80 °C. DNA was extracted from vaginal swabs using the PowerSoil DNA Isolation Kit (MO BIO Laboratories, Carlsbad). HPV genotyping and 16S rRNA sequencing were performed using DNA samples extracted from vaginal swabs using the Linear Array HPV Genotyping Test (Roche, Indianapolis, IN) and the MiSeq Platform (Illumina, San Diego, CA), respectively, as detailed previously^24^. Levels of 22 cytokines and chemokines were determined in CVL aliquots using multiplex cytometric bead arrays or enzyme-linked immunosorbent assays as described previously^24^. The genital inflammatory score system used in this study was described previously^24^. Briefly, levels of seven cytokines (IL-1α, IL-1β, IL-8, MIP-1β, MIP-3α, RANTES, and TNFα) were used to determine inflammatory scores; patients were assigned one point for each mediator when the level was in the upper quartile. Genital inflammation was defined as having at least 5/7 of the cytokines in the upper quartile.

### Measurement of cancer biomarkers

Levels of 24 cancer biomarkers: AFP, CA 15-3, CA 19-9, CA 125, CEA, CYFRA 21-1, sFas, sFasL, FGF2, β-hCG, HE4, HGF, IL-6, IL-8, leptin, MIF, OPN, prolactin, total prostate specific antigen (PSA), SCF, TGF-α, TNFα, TRAIL and VEGF were determined in CVL aliquots using the Milliplex MAP Human Circulating Cancer Biomarker Magnetic Bead Panel (Millipore, Billerica, MA) in accordance with the manufacturer’s protocol. A four-parameter logistic regression curve fit was used to determine the concentration. All samples were assayed in duplicate. The concentration values above the detection limit were substituted with the maximum observation values of the target and values below the detection limit were substituted with 0.5 of the minimum detectable concentration provided in manufacturer’s instructions. The log transformation was applied to normalize the data.

### Receiver operating characteristics analysis

The ROC analysis was performed to identify cancer biomarkers that discriminate appropriate patient groups. The strength of discriminators was measured with area under the curve (AUC) values. Cancer biomarkers with AUC greater than 0.8 or 0.9 were considered as good or excellent discriminators, respectively. The ROC analysis was performed using GraphPad Prism (version 5.0) software.

### Principal component analysis

The PCA was performed to reduce the observed variables into a smaller number of principal components (artificial variables) that will account for most of the variance in the observed variables. For the first two components, the difference among groups was assessed using the multivariate analysis of variance (MANOVA) model. If the overall difference was significant (*P* < 0.05), pairwise comparisons with Tukey adjustment were performed. The statistical differences for individual components were assessed using an analysis of variance (ANOVA) model.

### Correlation analysis

The Spearman’s rank correlation analysis was performed to investigate association of cancer biomarkers. A correlation matrix was computed using Spearman’s rank correlation coefficients (ρ) with *P* values, and graphically presented as a heat map. A *P* < 0.05 was considered significant.

### Mixed effects modeling

Linear mixed effects models were used to evaluate associations of cancer biomarkers with patient groups, adjusted for the random effect of replicates. The β-coefficient and standard error were estimated to show the direction of the association. If the overall difference was significant (*P* < 0.05), pairwise comparisons were performed with Tukey adjustment. Comparisons were adjusted for BMI, age and ethnicity in the linear mixed effects models by including these variables as predictors in the models, in addition to indicators for the patient groups (with Ctrl HPV− as the reference group). The β-coefficients therefore represent the expected change in each immune mediator for that patient group relative to Ctrl HPV− for women with the same values of the clinical covariates. Preliminary analyses demonstrated that there were statistically significant interactions between *Lactobacillus* dominance and the patient groups on the levels of the cancer biomarkers (*P* < 0.05). For these cancer biomarkers, comparison between the groups was performed separately for patients with LD microbiota and NLD microbiota.

### Hierarchical clustering analysis

The hierarchical clustering analysis was performed to show relationships between cancer biomarkers, cytokine/chemokine levels and metadata available for each patient, i.e. ethnicity, vaginal pH, *Lactobacillus* dominance, inflammatory scores. Prior to clustering, log-transformed concentration levels for each cytokine/chemokine and cancer biomarker were mean centered and then variance was scaled. Hierarchical clustering was performed using CIMminer based on Euclidean distance between rows and columns and average linkage cluster algorithm^64^.

### Other statistical analyses

Statistical differences in the demographic variables, HPV status and risk profile, and vaginal pH between patient groups were tested using ANOVA for continuous variables and Fisher’s exact test for categorical variables. Following the hierarchical clustering analysis, statistical differences in patient groups, *Lactobacillus* dominance, level of vaginal pH or inflammatory scores were tested using Kruskal-Wallis or Fisher’s exact test. All statistical analyses were performed using SAS 9.4.

## Supplementary information

Supplementary material
