# Supplementary material for: Features of the cervicovaginal microenvironment drive cancer biomarker signatures in patients across cervical carcinogenesis
Source: Sci Rep. 2019 May 14;9:7333. doi: 10.1038/s41598-019-43849-5 (PMC6517407; doi:10.1038/s41598-019-43849-5)
Supplement: Supplementary file 1 — Supplementary material [file 41598_2019_43849_MOESM1_ESM.pdf]

Supplementary information

Features of the cervicovaginal microenvironment drive cancer biomarker signatures in patients across cervical carcinogenesis

Paweł Łaniewski[1], Haiyan Cui[2], Denise J. Roe[2], Dominique Barnes[3,4], Alison Goulder[1], Bradley J. Monk[3,4,5,6], David L. Greenspan[3,4,5], Dana M. Chase[2,3,4,5,6], Melissa M. Herbst-Kralovetz[1,2,5,*]

[1]Department of Basic Medical Sciences, College of Medicine-Phoenix, University of Arizona, Phoenix, AZ, USA, [2]UA Cancer Center, University of Arizona, Tucson/Phoenix, AZ, USA, [3]Maricopa Integrated Health Systems, Phoenix, AZ, USA, [4]Dignity Health St. Joseph's Hospital and Medical Center, Phoenix, AZ, USA, [5]Department of Obstetrics and Gynecology, College of Medicine-Phoenix, University of Arizona, Phoenix, AZ, USA, [6]US Oncology, Phoenix, AZ, USA

* Correspondence:
Melissa M. Herbst-Kralovetz
Telephone: (602) 827-2247
Fax: (602) 827-2127
Email: mherbst1@email.arizona.edu

Supplementary table S1
Supplementary figures S1-S7

Supplementary table S1. Prevalence of HPV genotypes detected among the patient groups.

	n (%))	Ctrl HPV+ (n=11)	LSIL (n=12)	HSIL (n=27)	ICC (n=10)
**High-risk genotypes**					
HPV16	43 (72.88%)	9 (81.82%)	8 (72.73%)	19 (70.37%)	7 (70.00%)
HPV18	6 (10.17%)	0 (0.00%)	1 (9.09%)	4 (14.81%)	1 (10.00%)
HPV31	13 (22.03%)	0 (0.00%)	4 (36.36%)	9 (33.33%)	0 (0.00%)
HPV33	2 (3.39%)	0 (0.00%)	0 (0.00%)	1 (3.70%)	1 (10.00%)
HPV35	0 (0.00%)	0 (0.00%)	0 (0.00%)	0 (0.00%)	0 (0.00%)
HPV39	1 (1.69%)	0 (0.00%)	1 (9.09%)	0 (0.00%)	0 (0.00%)
HPV45	13 (22.03%)	1 (9.09%)	4 (36.36%)	7 (25.93%)	1 (10.00%)
HPV51	3 (5.08%)	0 (0.00%)	1 (9.09%)	1 (3.70%)	1 (10.00%)
HPV52	5 (8.47%)	0 (0.00%)	0 (0.00%)	3 (11.11%)	2 (20.00%)
HPV56	1 (1.69%)	0 (0.00%)	1 (9.09%)	0 (0.00%)	0 (0.00%)
HPV58	10 (16.95%)	4 (36.36%)	3 (27.27%)	3 (11.11%)	0 (0.00%)
HPV59	4 (6.78%)	1 (9.09%)	1 (9.09%)	2 (7.41%)	0 (0.00%)
HPV68	2 (3.39%)	0 (0.00%)	0 (0.00%)	1 (3.70%)	1 (10.00%)
**Low-risk genotypes**					
HPV6	3 (5.08%)	1 (9.09%)	0 (0.00%)	0 (0.00%)	2 (20.00%)
HPV11	0 (0.00%)	0 (0.00%)	0 (0.00%)	0 (0.00%)	0 (0.00%)
HPV26	0 (0.00%)	0 (0.00%)	0 (0.00%)	0 (0.00%)	0 0.00%)
HPV40	0 (0.00%)	0 (0.00%)	0 (0.00%)	0 (0.00%)	0 (0.00%)
HPV42	1 (1.69%)	0 (0.00%)	1 (9.09%)	0 (0.00%)	0 (0.00%)
HPV53	2 (3.39%)	0 (0.00%)	1 (9.09%)	1 (3.70%)	0 (0.00%)
HPV54	0 (0.00%)	0 (0.00%)	0 (0.00%)	0 (0.00%)	0 (0.00%)
HPV55	0 (0.00%)	0 (0.00%)	0 (0.00%)	0 (0.00%)	0 (0.00%)
HPV61	1 (1.69%)	0 (0.00%)	0 (0.00%)	0 (0.00%)	1 (10.00%)
HPV62	4 (6.78%)	3 (27.27%)	0 (0.00%)	1 (3.70%)	0 (0.00%)
HPV64	0 (0.00%)	0 (0.00%)	0 (0.00%)	0 (0.00%)	0 (0.00%)
HPV66	4 (6.78%)	2 (18.18%)	2 (18.18%)	0 (0.00%)	0 (0.00%)
HPV67	1 (1.69%)	0 (0.00%)	0 (0.00%)	1 (3.70%)	0 (0.00%)
HPV69	0 (0.00%)	0 (0.00%)	0 (0.00%)	0 (0.00%)	0 (0.00%)
HPV70	1 (1.69%)	1 (9.09%)	0 (0.00%)	0 (0.00%)	0 (0.00%)
HPV71	1 (1.69%)	0 (0.00%)	0 (0.00%)	1 (3.70%)	0 (0.00%)
HPV72	0 (0.00%)	0 (0.00%)	0 (0.00%)	0 (0.00%)	0 (0.00%)
HPV73	1 (1.69%)	1 (9.09%)	0 (0.00%)	0 (0.00%)	0 (0.00%)
HPV81	1 (1.69%)	1 (9.09%)	0 (0.00%)	0 (0.00%)	0 (0.00%)
HPV82	2 (3.39%)	0 (0.00%)	0 (0.00%)	2 (7.41%)	0 (0.00%)
HPV83	3 (5.08%)	0 (0.00%)	2 (18.18%)	1 (3.70%)	0 (0.00%)
HPV84	4 (6.78%)	1 (9.09%)	0 (0.00%)	3 (11.11%)	0 (0.00%)
HPV CP6108	2 (3.39%)	0 (0.00%)	0 (0.00%)	2 (7.41%)	0 (0.00%)
HPV IS39	0 (0.00%)	0 (0.00%)	0 (0.00%)	0 (0.00%)	0 (0.00%)

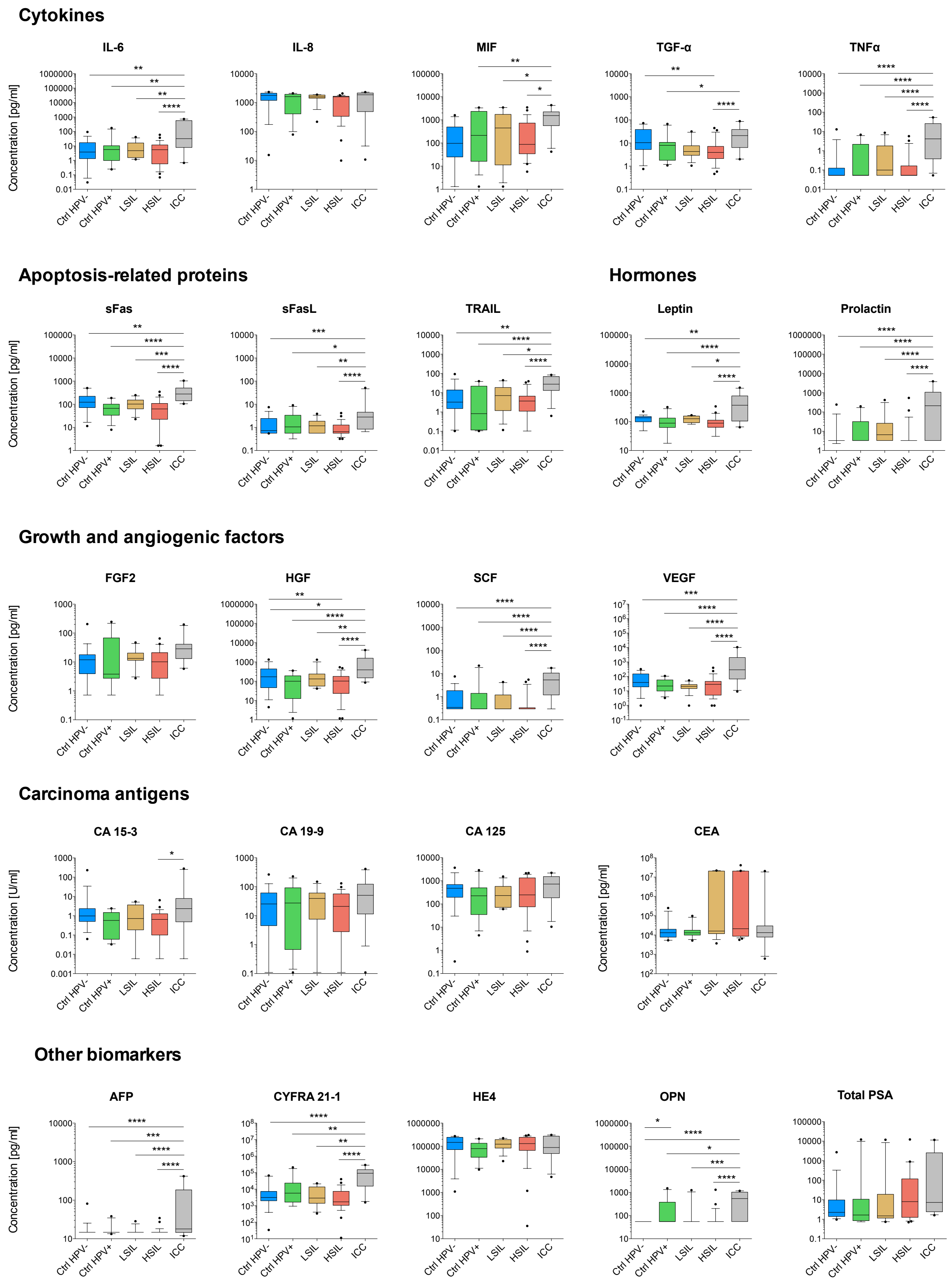

Supplementary figure S1. Levels of all tested cancer biomarkers in cervicovaginal lavages among the patient groups. Box-and-whiskers plots represent the median and interquartile range with whiskers ranging between the 10th and 90th percentiles; dots indicate outliers. *P* values were calculated using linear mixed effects models where group was the fixed effect and replicate was the random effect with Tukey adjustment. * *P*<0.05; ** *P*<0.01; *** *P*<0.001; *** *P*<0.0001.

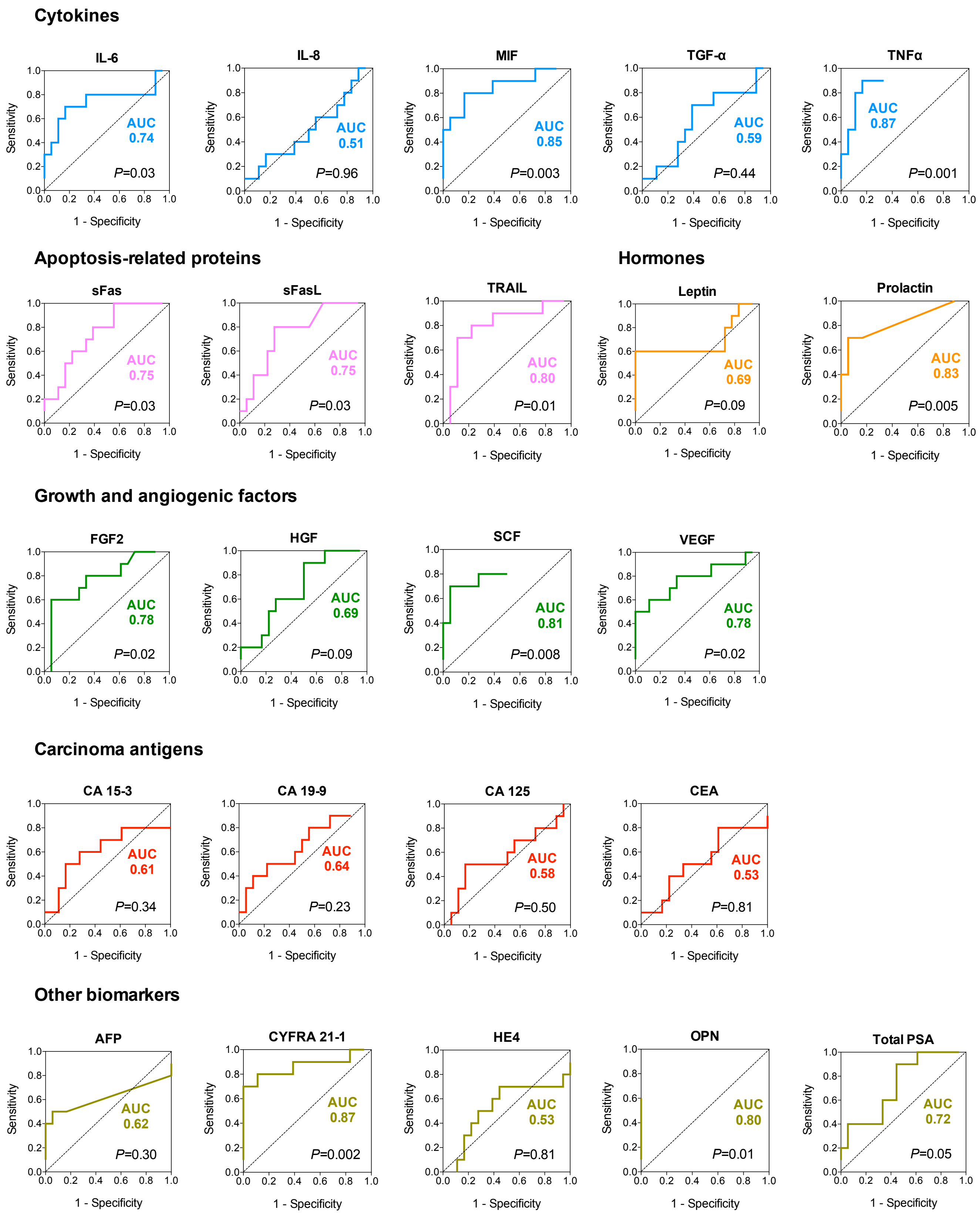

Supplementary figure S2. The receiver operating characteristics (ROC) analysis comparing ICC to Ctrl HPV– groups. ROC curves with areas under curves (AUC) and *P* values of all tested cancer biomarkers are shown. AUC greater than 0.8 indicates a good discriminator.

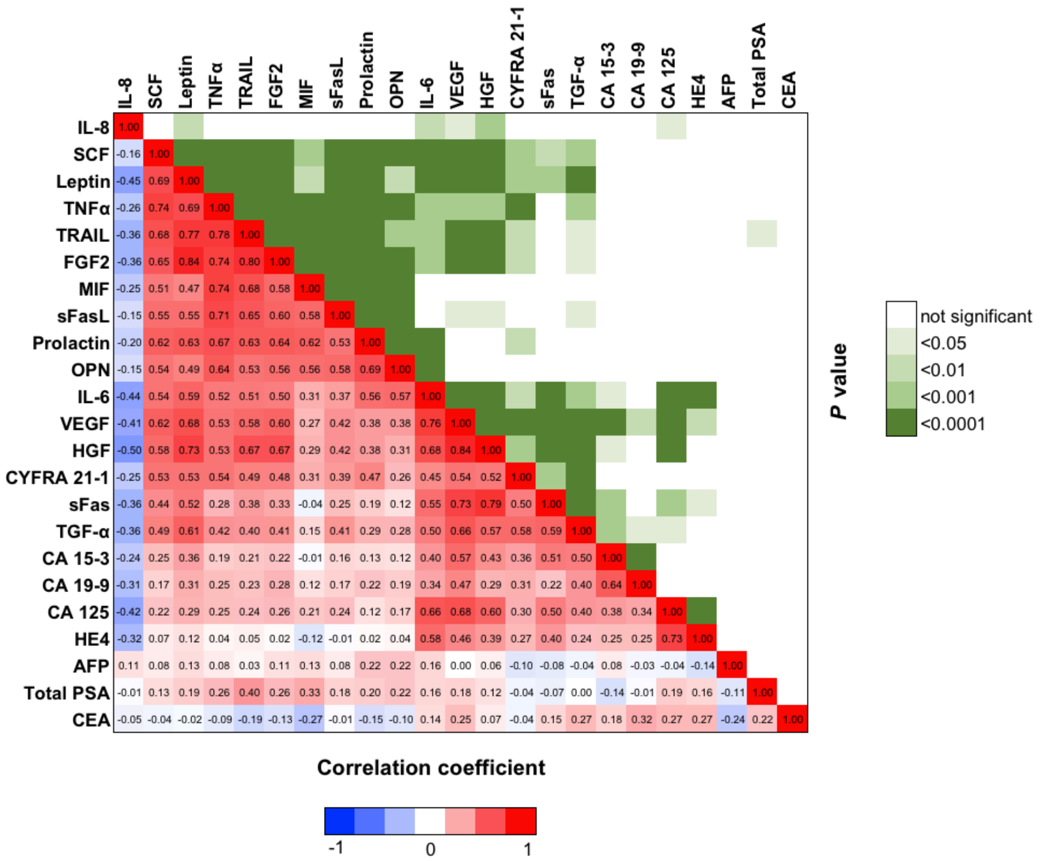

Supplementary figure S3. Correlation of cancer biomarkers to other cancer biomarkers in the cervicovaginal lavages among all the patients. Correlation coefficients (ρ) were calculated using Spearman's rank correlation analysis. Hierarchical clustering of correlation coefficients was performed using CIMminer based on Euclidean distance and average linkage cluster algorithm. A heat map shows Spearman's rank correlation coefficients in the lower left triangle and *P* values in the upper right triangle. Red and blue squares indicate positive and negative correlations, respectively, whereas green squares depict *P* values.

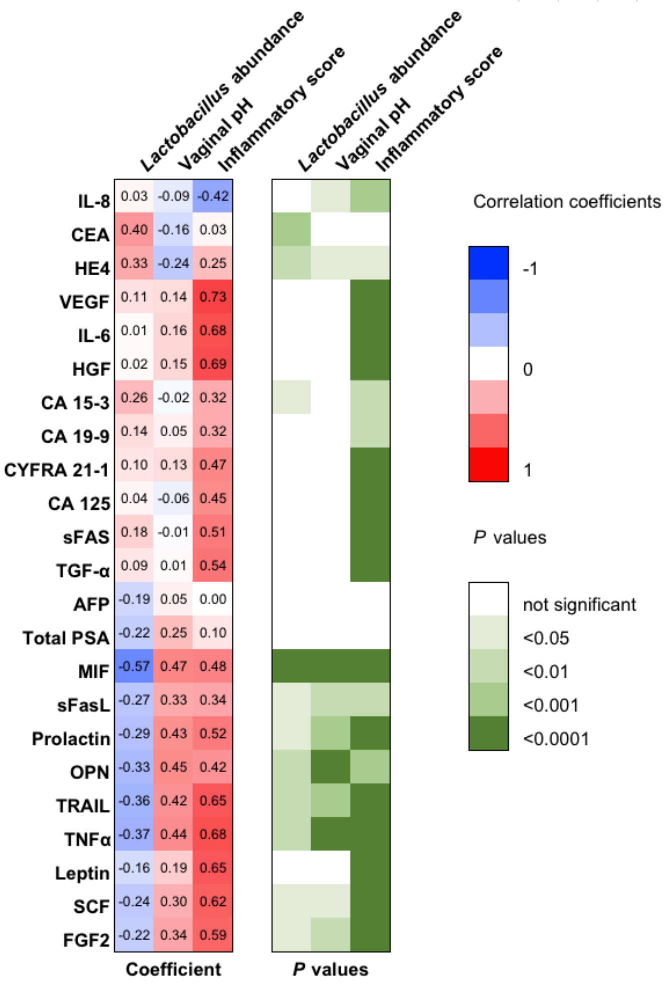

Supplementary figure S4. Correlation between levels of cancer biomarkers and *Lactobacillus* abundance, level of vaginal pH or inflammatory scores in cervicovaginal lavages among all the patients. Correlation coefficients (ρ) were calculated using Spearman's rank correlation analysis. Hierarchical clustering of correlation coefficients was performed using CIMminer based on Euclidean distance and average linkage cluster algorithm. Heat maps show Spearman's rank correlation coefficients on the left panel and *P* values on the right panel. Red and blue squares indicate positive and negative correlations, respectively, whereas green squares depict *P* values.

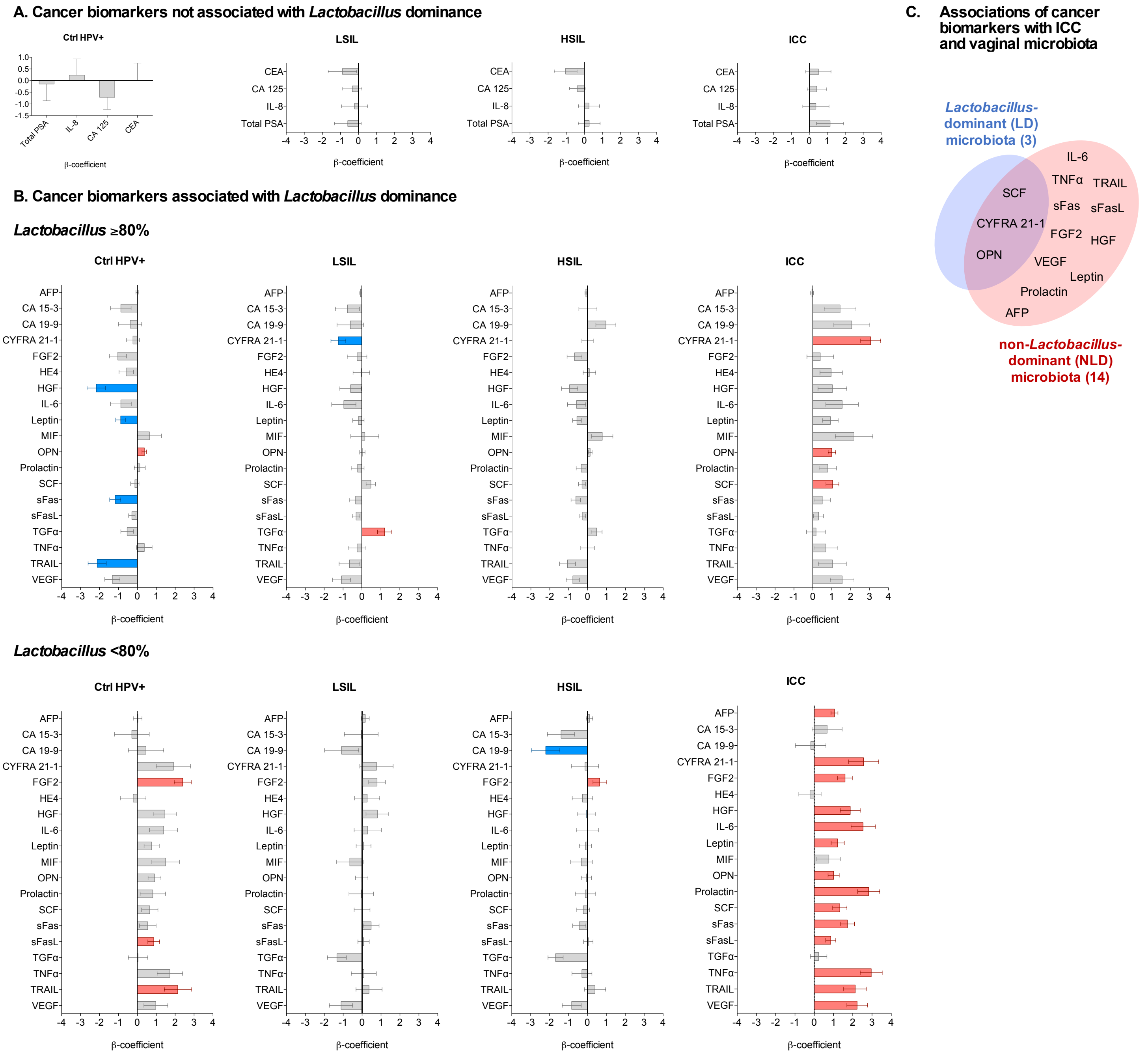

Supplementary figure S5. Associations of cancer biomarkers with patient groups after adjusting for *Lactobacillus* dominance, age, BMI, and ethnicity. A linear mixed effects model analysis of cancer biomarkers among patient groups is shown. β-coefficients of cancer biomarkers that were not associated with (**A**) or associated with *Lactobacillus* dominance (defined as ≥80% relative abundance) are depicted (**B**). Bars indicate β-coefficients of linear regression analysis; error bars represent standard error (SE). Red and blue bars indicate positive or negative associations, respectively, that were significant compared to Ctrl HPV- after adjusting for covariates (*P*<0.05). **C.** Venn diagram showing relationships between associations of cancer biomarkers with ICC and vaginal microbiota composition is depicted.

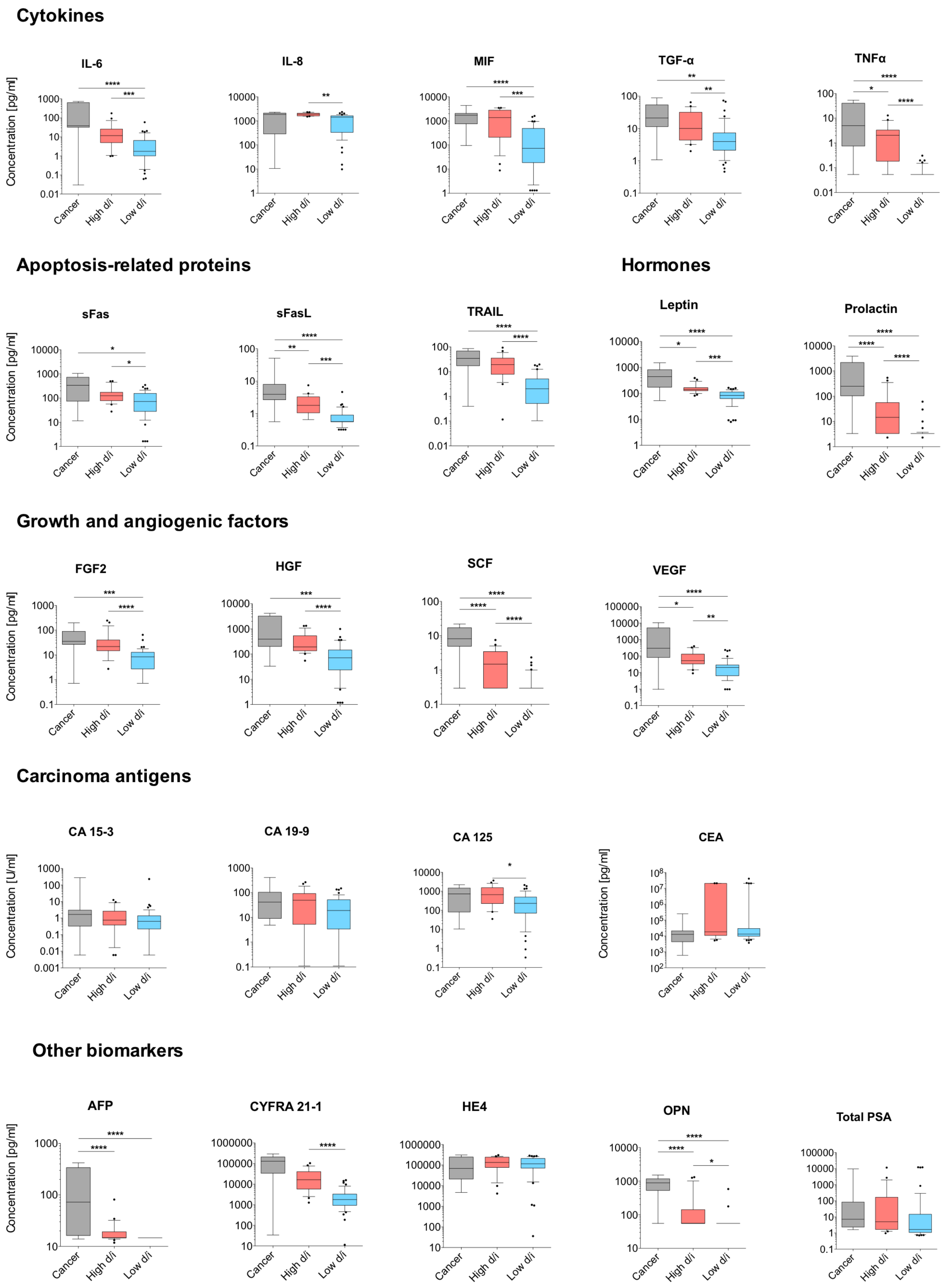

Supplementary figure S6. Levels of all tested cancer biomarkers in cervicovaginal lavages among the patient clusters: cancer-associated, high diversity/inflammation and low diversity/inflammation. Box-and-whiskers plots represent the median and interquartile range with whiskers ranging between the 10th and 90th percentiles; dots indicate outliers. Cancer: cancer-associated cluster; high d/i: high diversity/inflammation cluster; low d/i: low diversity/inflammation cluster. *P* values were calculated using linear mixed effects models where group was the fixed effect and replicate was the random effect with Tukey adjustment. * *P*<0.05; ** *P*<0.01; *** *P*<0.001; *** *P*<0.0001.

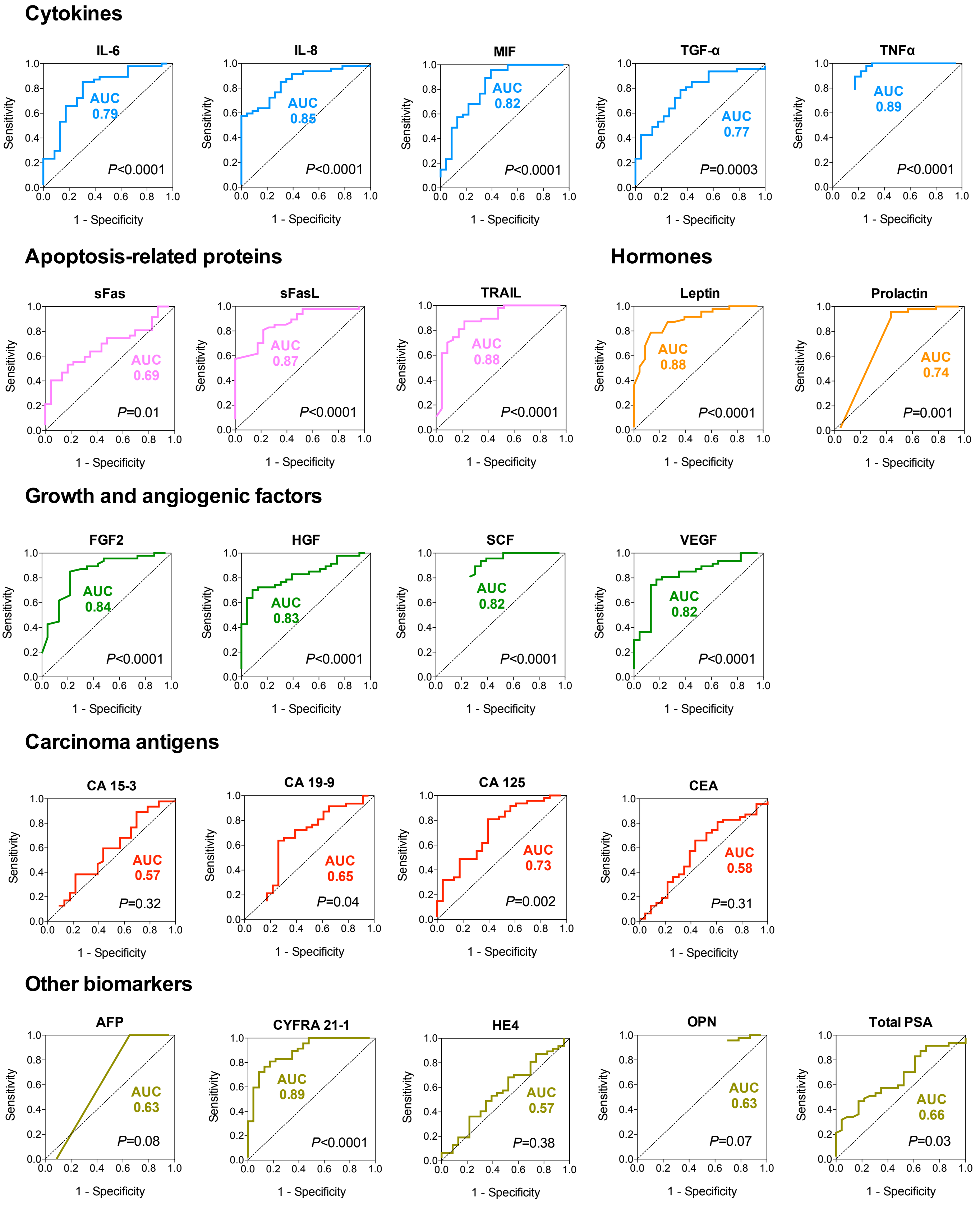

Supplementary figure S7. The receiver operating characteristics (ROC) analysis comparing patients from high and low diversity/inflammation clusters. ROC curves with areas under curves (AUC) and *P* values of all tested cancer biomarkers are shown. AUC greater than 0.8 indicates a good discriminator.
